# The association between municipal pandemic response and COVID-19 contacts to emergency primary health care services: an observational study

**DOI:** 10.1186/s12913-023-09489-2

**Published:** 2023-05-12

**Authors:** Vivian Midtbø, Ingrid Hjulstad Johansen, Steinar Hunskaar

**Affiliations:** 1 National Centre for Emergency Primary Health Care, NORCE Norwegian Research Centre AS, NO 5838, Box 22, Bergen, Norway; 2 Department of Global Public Health and Primary Care, Faculty of Medicine, University of Bergen, NO 5020, Box 7804, Bergen, Norway

**Keywords:** Emergency primary care, COVID-19, Pandemic, Out-of-hours service

## Abstract

**Background:**

Norwegian municipalities had diverse strategies for handling tasks related to the COVID-19 pandemic. The emergency primary health care services were involved to different extents. The aim of this study was to describe how contacts with the emergency primary health care service were affected by the pandemic, in terms of patient contacts related to COVID-19, prioritisation and first actions taken, and to analyse differences between the services.

**Methods:**

In this observational study, patient contacts to seven emergency primary health care services, from January 2020 to June 2021, were analysed. Descriptive analyses were applied. Data on the seven services’ involvement in the municipal pandemic response, in relation to testing the inhabitants for COVID-19, were collected.

**Results:**

There were 145 685 registered patient contacts within the study period. In total, 24% (*n* = 35,563) of the contacts were related to COVID-19, varying from 16 to 40% between the seven services. Of the COVID-19 related contacts, 96% (*n* = 34,069) were triaged to the lowest urgency level (range 76–99%) and 66% (*n* = 23,519) were patients contacting the services in order to be tested for COVID-19 (range 5–88%). The number of COVID-19 related contacts were unrelated to the number of confirmed COVID-19 cases among the inhabitants of the respective municipalities. The burden of COVID-19-related contacts mainly reflected the services’ involvement in COVID-19 testing as part of the municipal pandemic response.

**Conclusions:**

During the COVID-19 pandemic, several of the emergency primary health care services were assigned new tasks, such as being part of the municipalities’ system for carrying out testing for COVID-19. This had a major impact on their activity level. In the preparation for future pandemics, it should be discussed to which extent such use of the emergency primary health care system is appropriate, as additional tasks might affect the services’ preparedness to provide urgent medical care among the inhabitants.

**Supplementary Information:**

The online version contains supplementary material available at 10.1186/s12913-023-09489-2.

## Background

When the COVID-19 pandemic hit, several countries managed to re-organise their primary health care service to cope with the initial challenges caused by the pandemic, such as the lack of personal protective equipment or preventing spread of the virus by limiting physical consultations [1–5]. Extended use of telephone triage and telephone consultations, video consultations, and rapid development of tools to separate suspected COVID-19 cases from other conditions, were among the methods established [1, 2, 5–7].

The COVID-19 pandemic had a major impact on the utilisation of health care services. Initially, an increase in contacts to the emergency services due to the public’s worry and need for information about the new virus was observed [7, 8]. However, in a systematic review Moynihan et. al. found that health care utilisation decreased by a third during the first months of the pandemic, compared with the prepandemic period [9]. In the Netherlands there was seen a decrease in the use of out-of-hours services, especially among young persons [7]. The decrease in health care utilisation during the first months of the pandemic can be explained by the fear of becoming infected by COVID-19, a belief that the health care services were overloaded, but also by social distancing leading to fewer contagious infections. Furthermore, the public was advised to avoid contacting the health care services when possible [2, 7, 10].

When the COVID-19 pandemic reached Norway in the end of February 2020, the preparedness, and the premises for handling a pandemic, varied between the different municipalities and emergency primary health care services [5]. On the 12^th^ of March the Government initiated a social lockdown of the country, and during the same week the amount of telephone calls to some of the emergency primary health care services exceeded the capacity of the operators answering the telephone calls, resulting in hours of waiting to get through. Designated COVID-19 telephone lines were soon established by the municipalities, releasing some of the pressure on the emergency primary health care services. Because of limited supplies of test equipment and the risk of further spreading the virus from the person being tested to the health personnel, there were strict criteria for testing for COVID-19 during the first months of the pandemic, and a request or a referral from a medical doctor was required [11]. To restrict transmission of the virus, separate infection rooms were established in most of the emergency primary health care services, and many services also established designated airway clinics [5].

A large part of the strategy to handle the pandemic in Norway was through the strategy of “testing, isolation, contact tracing, and quarantine”, which was mainly managed by the municipalities [12]. The emergency primary health care services were involved to different extents. Some of the services were given the responsibility for carrying out testing for COVID-19 for all the inhabitants in their district and some were responsible for airway clinics, handling patients with COVID-19 or symptoms consistent with COVID-19.

The aim of the present study was to describe how patient contacts to the emergency primary health care services were affected by the COVID-19 pandemic, in terms of patient contacts related to COVID-19, urgency degrees, and first actions taken. We also analysed how the different ways of organizing the pandemic response within the municipalities affected the load on the emergency primary health care service.

## Methods

This was an observational study, analysing data from seven emergency primary health care services, during the period January 2020 to June 2021.

### Setting

Norway has a two-layered health care system where the primary health care service, which is managed by the municipalities, has a gate keeping function for secondary health care services, which is managed by the state. The municipalities are obliged to provide emergency primary health care service 24/7 and the inhabitants are advised to contact the service in urgent situations when their regular general practitioner (GP) is not available. Telephone is the most common mode of contact and the national number 116 117 routes the call to the nearest local emergency medical communication centre. The operators handling the telephone calls are primarily trained as registered nurses. The operators triage the patients and refer them to the most appropriate level of care. In non-urgent cases, in which a consultation with a GP is not indicated, the operator may handle the contact solely by telephone consultation, providing self-care advice and/or advising the patient to contact his or her GP within office hours.

The data for this study was collected within a sentinel network called the Watchtower project, consisting of seven Norwegian emergency primary health care services (the watchtowers), which have registered data on all patient contacts to their services since 2006 [13]. The watchtowers were originally included in the project by a set of selection criteria, designed to ensure a sample as representative as possible for the Norwegian municipalities as a whole. The seven services reflected different organisational models for emergency primary health care services. Furthermore, the distribution of age, gender, degree of centralisation of the population in the municipalities, distribution of employment by branches of business and industry, the municipality’s public economy, and also gross income among men were considered in the selection process [13]. Combined, the seven watchtowers cover 4.6% of the Norwegian population, but each service vary regarding the number of inhabitants covered, the smallest covering about 5000 inhabitants and the largest covering about 97 000 inhabitants.

### Data collection and variables

Information about all contacts to the participating clinics, both by telephone and direct attendance, was recorded anonymous in an online database (Zoho Creator®) by the personnel on duty. Among the collected variables, we used the following variables in the current study:

*Contacts related to COVID-19* was defined as contacts about disease / symptoms, spread of the virus or worries related to the coronavirus.

*Action taken*: Telephone consultation by operator, medical consultation by a doctor, and COVID-19-test (PCR-test), which was added as a possible option in the database from 20^th^ of March 2020.

*Time of day* was registered by three categories: Daytime 08.00–15.29, evening 15.30–22.59, and night 23.00–07.59.

*Urgency level* was assessed by the operator in accordance with the Norwegian Index for Medical Emergency Assistance [14] into one of three urgency levels defined by colour: Red (acute), yellow (urgent) and green (non-urgent).

We collected information about the watchtowers’ involvement in the municipal pandemic response, in relation to testing the inhabitants for COVID-19.

The monthly numbers of COVID-19 cases confirmed by a PCR-test reported to the Norwegian Surveillance System for Communicable Diseases (MSIS) were obtained for the population belonging to each watchtower district [15].

### Data analysis

We performed descriptive analyses of the data material month by month, to identify how patient contacts, including contacts related to COVID-19, developed during the study period. The total numbers of patient contacts, and actions taken in terms of medical consultation by a GP, telephone consultation by operator and testing for COVID-19, were counted for every month together with the numbers of contacts registered within the different urgency categories. Furthermore, we analysed data from each watchtower separately to be able to compare the different services in relation to their share of contacts related to COVID-19 throughout the study period. StataSE 17 was used to analyse the data.

## Results

### Patient contacts to the watchtowers

During the study period there were a total of 145 685 patient contacts to the seven watchtowers, 35 563 (24%) of these were contacts related to COVID-19.

**Fig. 1:**
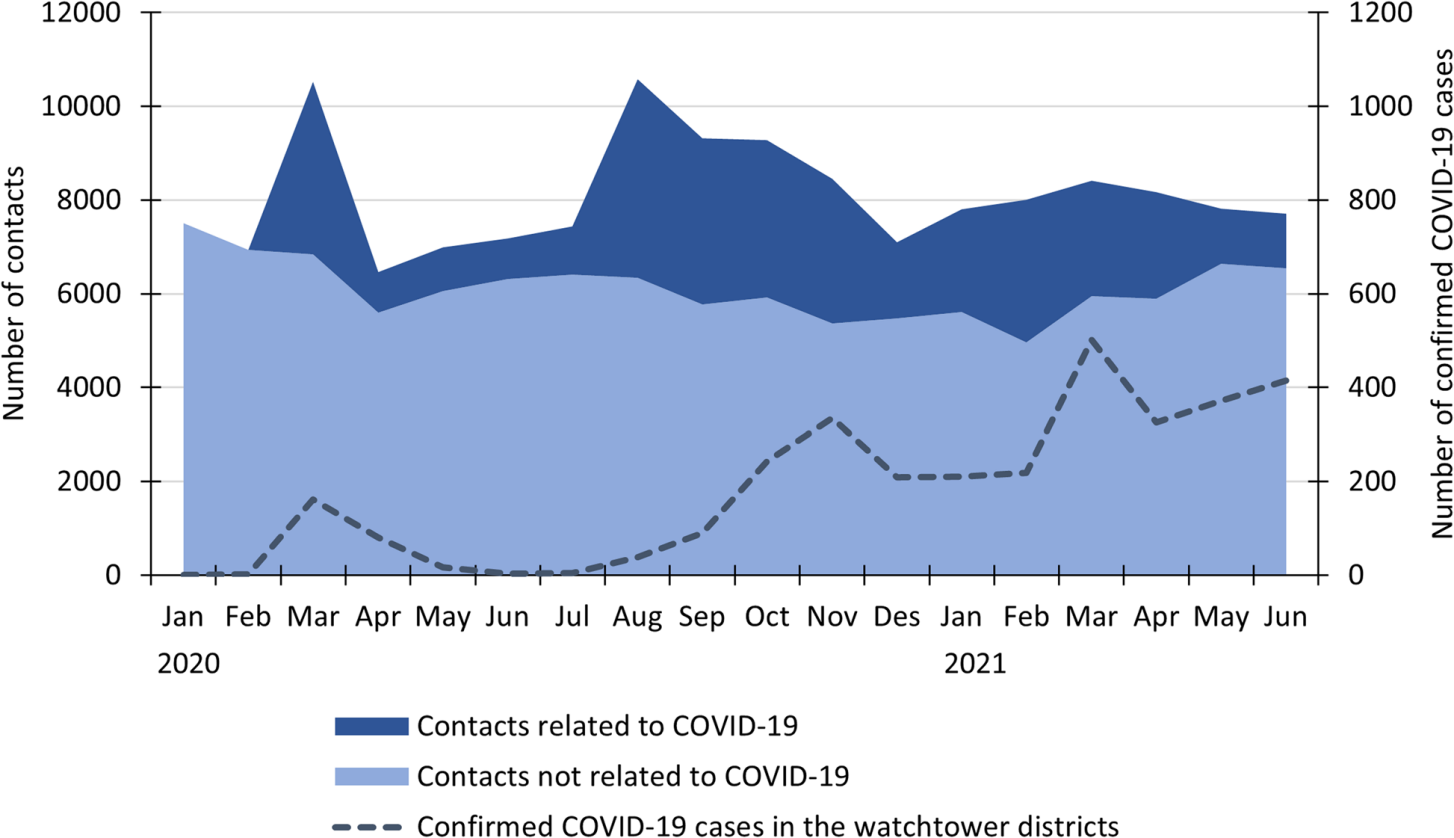
Number of contacts to all the watchtowers combined, shown by contacts related to COVID-19, contacts not related to COVID-19, and the number of COVID-19 cases confirmed by a PCR-test reported to the Norwegian Surveillance System for Communicable Diseases (MSIS) in the municipalities covered by the watchtowers, month by month from January 2020 to June 2021

There were two distinct peaks of contacts related to COVID-19 during the study period (Fig. 1). The first peak was in March 2020, when the Government initiated a lockdown of the country. 35% (*n* = 3678) of the total contacts to the watchtowers during the first month of the pandemic were related to COVID-19, varying from 16 to 40% between the seven watchtowers. The week when the lockdown was initiated, 51% (range 30–57%) of the total contacts to the watchtowers were related to COVID-19.

The second peak, in August 2020, was related to a change in the criteria for testing. The criteria for testing became much more liberal and testing should be available to anyone who suspected that they could be infected. This month there were a total of 4233 contacts related to COVID-19. There were, however, only 38 confirmed COVID-19 cases among the inhabitants in the seven watchtowers (Fig. 1), which implies that numbers of COVID-19 related contacts were not related to the numbers of confirmed COVID-19 cases.

### Urgency levels

**Fig. 2:**
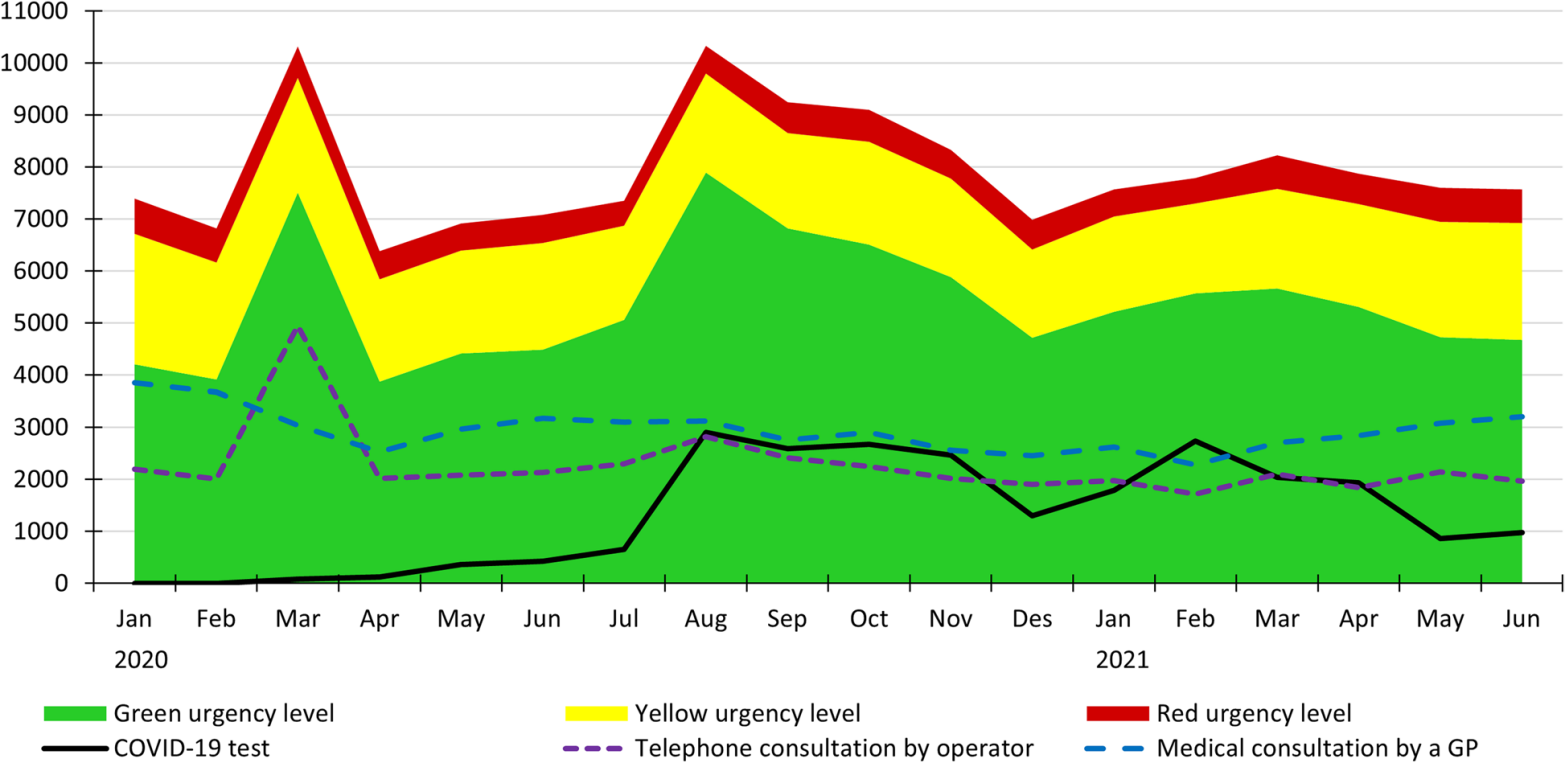
The total number of contacts per month resulting in telephone consultation by operator, a consultation by a GP, and a COVID-19 test, and the number of total contacts triaged to green, yellow, and red urgency level, throughout the study period

The number of contacts triaged to yellow or red urgency levels were stable throughout the study period, while there were large variations in the number of contacts triaged to green urgency level (Fig. 2). In total, 96% (range 76–99%) of the contacts related to COVID-19 during the study period, were triaged to green urgency level. In the watchtowers who had test responsibility for the whole study period the total percentage was 99%, while in the watchtowers not responsible for testing, the total percentage was 89%.

### Actions taken

Telephone consultation by operator had a large peak in the first month of the pandemic, when more than twice as many patient contacts received this action, compared with the month before and the month after (Fig. 2). Furthermore, during the week the lockdown was initiated, 57% (range 50–69%) of the total patient contacts to the watchtowers were handled by telephone consultation by operator. The increased number of telephone consultations by operator were seen during both day and evening shifts during the first weeks of the pandemic (data not shown).

Compared with the year before the pandemic hit (see Additional table 1), the number of contacts handled by a medical consultation by a GP were lower during all months of the pandemic period. March and April, which were the two first months of the pandemic, saw a reduction in medical consultations by a GP of respectively 24% in March (4010 consultations in 2019, 3040 in 2020) and 36% in April (3969 consultations in 2019, 2527 in 2020).

Testing for COVID-19 was quite low in numbers during the first months of the pandemic, due to limited supplies of test equipment, but increased from August 2020 (Fig. 2). In total, 66% (*n* = 23,519) of the contacts related to COVID-19 were due to inhabitants contacting the services to get tested for COVID-19 (range 5–88%). 93% of the COVID-19 tests were performed during day shifts.

### Different patterns of contacts related to COVID-19

**Fig. 3:**
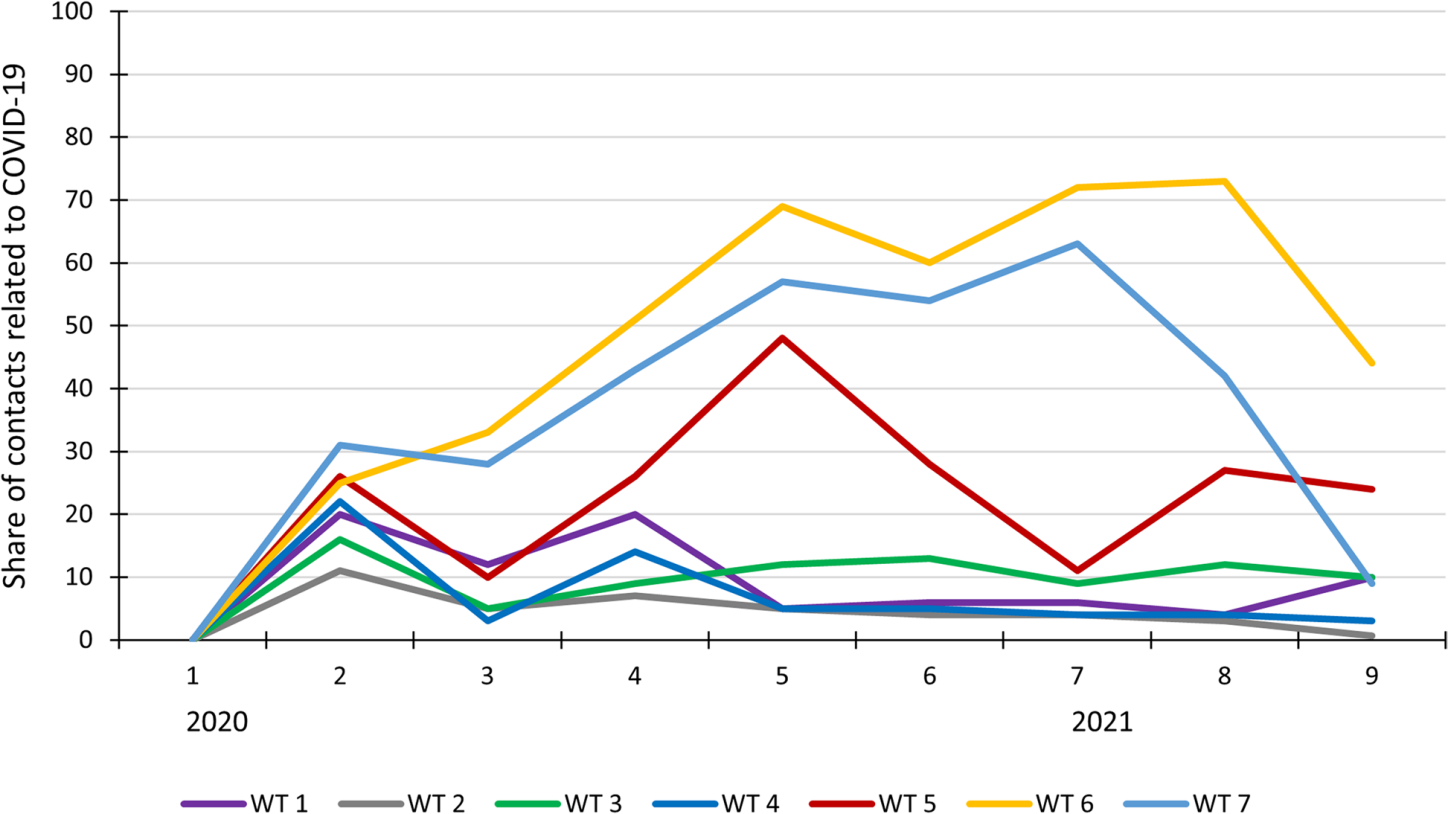
Share of contacts related to COVID-19 in each of the seven watchtowers (WT), shown by 9 periods of two months, from January 2020 to June 2021

In March 2020 all seven watchtowers had a peak in the share of contacts related to COVID-19 (range 16–40%). After this initial peak, there were both similarities and differences between the watchtowers regarding the share of contacts related to COVID-19 throughout the study period (Fig. 3).

**Fig. 4:**
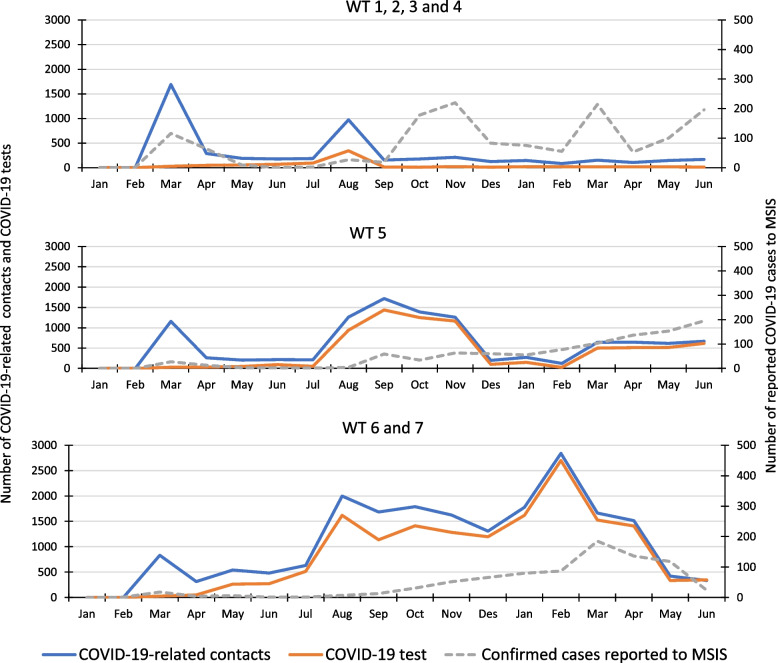
Three different patterns of contacts to the watchtowers (WT) related to COVID-19, from March 2020 to June 2021, the number of patient contacts handled by a COVID-19 test, and the number of COVID-19 cases confirmed by a PCR-test, reported to the Norwegian Surveillance System for Communicable Diseases (MSIS) in the municipalities covered by the watchtower districts

Three distinct patterns of contacts were identified (Fig. 4), which clearly reflected the services’ involvement in the municipalities’ system for testing the inhabitants for COVID-19.

Watchtowers 1, 2, 3 and 4 (Fig. 4, graph on the top) had no responsibility for testing inhabitants for COVID-19 after mid-August 2020 and they had relatively low numbers of contacts related to COVID-19 after the peak in August 2020. Even though the numbers of confirmed COVID-19 infections increased among the inhabitants covered by these services from September 2020, the numbers of COVID-19 related contacts did not increase (Fig. 4).

Watchtower 5 (Fig. 4, graph in the middle) had a similar pattern as watchtowers 1, 2, 3, and 4 until August 2020, but continued to have high numbers of contacts related to COVID-19, up to December 2020. This service was involved in testing until November 2020, and a large part of the COVID-19 related contacts were related to testing. Figure 4 shows that during the period when contacts related to COVID-19 peaked the most, the numbers of confirmed COVID-19 infections were still quite low.

Watchtowers 6 and 7 (Fig. 4, graph at the bottom) had high numbers of contacts related to COVID-19 during the whole study period. These services were responsible for COVID-19 testing of their inhabitants during the whole period. In August 2020 there were low numbers of confirmed COVID-19 cases among the inhabitants covered by these services (Fig. 4). Still, the number of COVID-19 related contacts were high and mostly related to testing.

## Discussion

Two distinct peaks of COVID-19 related contacts, caused by different mechanisms, were observed during the study period. During the first peak, a large number of the contacts were handled by telephone consultations by the operators. From the second peak throughout the rest of the study period, a large number of the COVID-19 related contacts were inhabitants contacting the emergency primary health care services in order to get tested for COVID-19. Most of the COVID-19 related contacts were triaged to green urgency level. There were both similarities and differences between the services in the share of contacts related to COVID-19, which reflected how involved they were in the municipalities’ system for COVID-19 testing. It was also evident that the number of confirmed COVID-19 cases in the MSIS among the inhabitants covered by the seven watchtowers were unrelated to the number of COVID-19-related patient contacts to the services.

The initial peak of COVID-19 related contacts in March 2020 seemed to be triggered by a vast need for information among the public. When the Government initiated a social lockdown of the country on March 12^th^ [11], an extraordinary number of contacts to the emergency primary health care services were observed. In our data material, a large part of these contacts was non-urgent and handled by telephone consultations by the operators. Similar findings have been reported elsewhere. A study from Italy reported that call volumes to an emergency medical service increased by 56% during the two weeks after the announcement of the first confirmed COVID-19 case and many callers were worried persons requesting information and guidance about the new illness [8]. Furthermore, in the Netherlands, an increase in consultations for COVID-19 like symptoms was observed in the beginning of the pandemic, which was described as likely being caused by fear and uncertainty concerning COVID-19 [7]. Islam et al. suggested in their study from the Belgian out-of-hours services [16] that operators might have been extra cautious in their dispatch decisions, leading to physical consultations with health care professionals. This could also be of relevance in our study, with regard to the large number of contacts handled by telephone consultation by the operators and the decrease in numbers of medical consultations by a GP.

Norway had a restrictive testing policy during the first five months of the pandemic [11]. The second time the COVID-19 related contacts to the watchtowers peaked, in August 2020, can be related to a change in the criteria for testing. The supplies of COVID-19 test equipment improved, and in August 2020 the municipalities should be able to provide testing for any person with suspected COVID-19 infection, without a referral from a medical doctor [11]. After the second peak of COVID-19 related contacts, in August 2020, a large part of the contacts related to COVID-19 were contacts to three of the watchtowers (WT 5, 6 and 7). These three watchtowers had a large responsibility in testing their inhabitants for COVID-19, which clearly affected the numbers of contacts related to COVID-19. A large share of the COVID-19 related contacts to these three services from August 2020 through the rest of the study period, was due to inhabitants contacting the services to be tested for COVID-19. Studies from other countries have documented decreased utilisation of health care services, including emergency services, during the pandemic compared to prepandemic [15, 17]. These services’ role in testing are unknown.

When comparing the number of confirmed COVID-19 cases reported to the MSIS with the COVID-19 related contacts to the watchtowers, it was evident that there was no association between the two. COVID-19 related contracts reflected how involved the emergency primary health care services were in the municipalities system for testing the inhabitants for COVID-19 and the politics related to criteria for testing, rather than the actual burden of disease in the municipalities.

In Norway, the municipalities were responsible for the whole strategy of “testing, isolation, contact tracing, and quarantine”. When the municipalities were instructed by the authorities to increase their test-capacity, this created an even more challenging situation for some municipalities because of an already strained test-capacity [12]. Already early in the pandemic, it was evident that the emergency primary health care services had the ability to adapt to the pandemic by acting quickly, being flexible, and altering the way they worked [5]. Also during previous pandemics, the primary health care service has demonstrated its ability to adapt to rapid changes, such as the increased patient demand during the influenza pandemic in 2009 [18]. Such flexibility could explain why many of the services became so involved in the COVID-19 testing regimes. In addition, the emergency primary health care services constituted existing infrastructures within the municipalities, with access to health care personnel 24/7. Adding new tasks to existing services was probably easier and less resource-demanding compared with developing a new service from scratch. However, the emergency primary health care service is an important part of the emergency medical service, with a core task to be available to inhabitants with acute or urgent medical needs. The involvement in testing could have affected the services’ preparedness. Norwegian emergency primary health care services were involved in handling the pandemic in different ways and extents. In some municipalities testing was organised separate from the emergency primary health care service, by establishing dedicated test stations for COVID-19 testing. In other municipalities, testing was handled by the GP offices. In the watchtowers that did not have test-responsibilities, considerably lower numbers of contacts related to COVID-19 were observed, compared with services with full test-responsibility.

### Strengths and limitations

The Watchtower project has been running since 2006, which means that the project is well integrated in the participating services. The overall quality of the data is good and the number of incomplete recordings during the study period of 2020 and 2021 were 3.6% and 3.4%, respectively [19]. There is an underreporting of cases in the Watchtower project. When comparing the estimated national number of GPs’ consultations based on the watchtower data with the yearly statistics on reimbursement claims from the services, there is a deviation between 6 and 28% in the years before the pandemic (2007–2019) [19]. Underreporting of cases can be due to several factors, such as busy shifts and periods with many temporary workers, for instance during holidays. The large volume of patient contacts to the services during some of the periods in the current study makes it reasonable to assume that some of the contacts have not been registered. Furthermore, in many cases, symptoms of COVID-19 and symptoms related to a common cold or influenza can be very similar, which may have complicated the task of registering contacts related to COVID-19. However, because of the large number of observations, we believe that our data provide a reliable and valid description of patient contacts to the emergency primary health care service in Norway during the pandemic.

## Conclusions

The emergency primary health care services have proven to be a safety net that the public turns to not only in cases of medical urgencies, but also when seeking advice or information in other events of uncertainty relating to health issues. During the pandemic the emergency primary health care services were assigned new tasks, such as being part of the municipalities system for carrying out testing for COVID-19. Their role in testing had a major impact on their activity level. In the preparation for future pandemics, it should be discussed to which extent such temporary use of the emergency primary health care services is appropriate, as additional tasks might affect the services’ preparedness to handle acute or urgent medical issues among the inhabitants.

## Supplementary Information

**Additional file 1.** Prepandemic numbers of patient contacts and actions taken.

## Data Availability

Data are available upon reasonable request to the corresponding author. The data set generated and analysed during the current study are not publicly available because the approvals from the ethics committee and the privacy ombudsman for research do not permit disclosure of raw data.

## Abbreviation

GP: General practitioner
