# Supplementary material for: The association between municipal pandemic response and COVID-19 contacts to emergency primary health care services: an observational study
Source: BMC Health Serv Res. 2023 May 12;23:479. doi: 10.1186/s12913-023-09489-2 (PMC10175054; doi:10.1186/s12913-023-09489-2)
Supplement: Supplementary file 1 — Additional file 1. Prepandemic numbers of patient contacts and actions taken. [file 12913_2023_9489_MOESM1_ESM.pdf]

Additional table: Prepandemic numbers of patient contacts and actions taken.

		Actions taken	
**Month 2019**	**Total patient contacts**	Medical consultation by a GP	Telephone consultation by operator
	**n**	**n**	**n**
Jan	7728	4321	2011
Feb	7223	3918	1931
Mar	7416	4010	2013
Apr	7298	3969	1942
May	7395	4209	1897
Jun	7115	3944	1844
Jul	7399	3953	2220
Aug	6891	3767	1969
Sep	6971	3862	1875
Oct	7268	3836	1971
Nov	7359	3914	2043
Des	8764	4605	2502
**Total**	**88827 (100%)**	**48308 (54%)**	**24218 (27%)**

Total numbers of patient contacts to the seven emergency primary health care services, and the number of contacts handled by medical consultation by a GP and by telephone consultation by operator, by each month in 2019.
